# Corruption – Taking a Deeper Dive: Comment on "We Need to Talk About Corruption in Health Systems"

**DOI:** 10.15171/ijhpm.2019.63

**Published:** 2019-09-02

**Authors:** Rakhal Gaitonde

**Affiliations:** Achutha Menon Centre of Health Science Studies, Sree Chitra Tirunal Institute of Medical Sciences and Technology, Kerala, India.

**Keywords:** Corruption, Systems Approach, Problematization, Multi-level Interventions

## Abstract

This commentary while agreeing broadly with the points raised by the editorial by McKee et al, seeks to broaden and deepen those arguments. The commentary contends that unless we understand corruption as deeply embedded in and propping up systems of power differentials, we will not be able to design interventions that will tackle corruption at its roots. The commentary further points to the context specific nature of corruption and hence the futility of attempting a single definition. This it contends will merely hide the deeper context specific causes. It calls for the using theoretical insights that draw from post-positivist approaches to enhance the conceptualization of corruption as systemic. Further it points to the importance of the underlying problematization of corruption in attempts to tackle it. It ends with a call for attempts at multiple levels with the broader aim of evolving caring and just systems of healthcare rather than focusing on narrow ‘politically feasible’ interventions.

The editorial by McKee et al,^1^ is a welcome call to recognizing corruption as a major issue in public health. It was especially welcome in that it acknowledges the complexity of the issue, and underlined the futility of simplistic and moralistic approaches to the issue. In this commentary, while broadly agreeing with the points raised by the authors, I would like to propose a few ways of building on the arguments put forward in the editorial.

In the opening paragraphs the authors point out to the fact that corruption is “our” dirty secret, referring to the health policy community.^1^ It is well known that the issue of corruption has plagued communities for decades now, and that there have been several attempts at its control at large and small scales. The interesting point however is the rise of corruption as a legitimate topic of discussion and research in the health policy community at this point in history. The editorial even notes that some consider corruption as merely a “neoliberal attack on the state” and thus not the best focus of research.^1^ This is a warning against simplistic solutions to corruption rather than a commentary on the legitimacy of the topic as such. If one considers corruption as emergent from regimes of governance, then simplistic law and order based solutions will unlikely make any impact.

The definition adopted by the Cochrane review^2^ tried to capture the complexity of the issue of corruption. It was very clear that no single and universal form of corruption was recognized. It was clear that the significance of individual acts varied depending on the circumstances and the contexts.

While a corrupt act is essentially transacted between two individuals, the justification and enabling of the act draws on larger macro level processes. Structural adjustments and the consequent contraction of public funding of the social sector for example create the eco-system for ‘survival corruption’ among front line public servants. While survival corruption makes working conditions marginally tolerable for the frontline workers, it adversely affects the access of marginalized communities who are dependent on public services to a greater extent.

Another example is the eco-system created by an unregulated market in the private health sector thanks to the “opening up” of a number of low- and middle-income country economies under pressure from International Financial Institutions. There is an over-supply of high end medical technology which creates the setting for “cut practice” (the practice of diagnostic centers paying doctors a fixed ‘kickback’ for referring patients to them) becoming a norm in the medical sector in India. The “cuts” offered to doctors for referrals creates an incentive for over investigation and irrational investigation, thus propping up the inflated market, but again at the cost of the patient who pays for this. What is also important is the way in which the individual corrupt practice draws attention away from underlying causes such as structural adjustments, contraction of public sector financing, and processes of privatization and influence of the medico-industrial complex.

Seen from this perspective, any attempt to evolve a universal definition of corruption at the level of practice has the danger of missing the nuance of historical and negotiated development of various practices and their significance in different settings. It is with this in mind that the definition evolved for the Cochrane review chose to stay at the level of principle. The lack of agreement on what exactly constitutes corruption, the first of five points the editorial makes,^1^ is not unexpected, and neither is it a crucial block to the efforts at controlling corruption as the authors suggest. Instead it is a pointer to corruption’s essentially context specific nature. In this framing – corruption may be seen as something that actually contributes to propping up a particular iniquitous systemic arrangement.

## Theoretical Approaches

One way of classifying theories of the policy process is to divide them into those that take a positivist approach and those that are post-positivist. One essential difference in the two are the way in which the problem the policy is addressing is seen. In positivist approaches to policy there is an underlying assumption of a single, obvious and unambiguously describable and measureable problem that is being addressed. In post-positivist approaches on the other hand the problem being studied are not seen as having any universal core essence, but rather posit the attribution of meaning to given situations in different contexts. Such approaches focus on what are termed the underlying ‘problematizations’ which refer to the way in which a given issue is being represented, the way it is being framed or constructed as a problem and the underlying assumptions that are essential to such a framing. In other words post-positivist approaches interrogate the way a problem is framed, the way it is legitimized as a problem in need of solving and thus taps into broader systems of meaning and discourse.

This sort of approach points to the fact that the “problem” of corruption is not a singular, uncontested “fact.” What type of problem corruption is represented be, will define the types of solutions that are suggested as being legitimate. Thus solutions that suggest increasing policing, stricter punishments, and ombudsmen as legitimate solutions seem to suggest corruption as a law and order problem with the need being to regulate errant providers.

Drawing on insights from such an approach then, the question is not only to identify particular “windows of opportunity,” but indeed to ask why these particular windows were opened at this point in time, and why only for particular types and forms of corruption and for particular types of solutions. In other words what makes some forms of corruption more “politically acceptable” to control than others. While these itself may be beneficial for the most marginalized communities who depend on these services, at least in the short run, we should not delude ourselves that such interventions will lead to long term and sustainable results.

Framing corruption among front line workers, without factoring in working conditions and poor pay (for example) is unlikely to make a lasting impact. In other words tackling the most visible or more peripheral of manifestations of a systemic issue without tackling the deeper underlying structural/design issues will potentially shift the manifestation of corruption to different spaces/forms.

## So What Is to Be Done?

Following the analysis and framing presented above means seeing corruption as a systemic feature rather than as an aberration. This means that approaches to tackle corruption would have to focus on developing locally tailored interventions born from a deeper understanding of local dynamics rather than focus on solutions that are universal or up-scalable. What then are the possible strategies to tackles corruption? If one starts from the framing presented above which sees corruption as a systemic issue, then it becomes obvious that measures to tackle corruption need to be at multi-levels and need to challenge deeply vested interests. For such processes then there needs to be mobilization not only at the community level, but also at the provider, policy-maker and the political decision-maker level. Indeed it needs to be appreciated that changes need to happen not only at the local behavior levels but indeed at the levels of norms, meanings and systems.

One of the potentially promising approaches documented in the Cochrane review is that of Kyrgystan, which simultaneously worked on both increasing transparency, increasing wages and making more funding available at the organizational level. This represents the various system level bottlenecks that were the basis of encouraging corrupt practices. Thus by combining increased transparency with better wages and increased flexible funding available at the institutional level, this represents a more nuanced and systemic understanding of corruption that has the potential of getting to the root of the issue. Coming in the aftermath of the collapse of the Soviet Union and the promises of alternative modes of governance, the Kyrgyz example highlights the importance of the historical and political specificity needed while tackling corruption.^4^

In India a few examples of such initiatives – though not necessarily explicitly articulated as anti-corruption initiatives as such – but which have significant promise in my opinion are the systematic initiatives building on the community-based monitoring and planning initiative in the state of Maharashtra.^5^ After the initial work on community-based accountability which focused on the rural public health system, the work expanded organically to include the private sector in both rural and urban areas and evolved a number of innovative strategies to make impacts not only on the behavioral level but also on the normative level.^6^ This included the convening of a number of Citizen–Doctor forums (see http://mypcdf.org/), as well as a network of ethical doctors called Alliance of Doctors for Ethical Healthcare (ADEH) (http://www.ethicaldoctors.org/). The ADEH has opened chapters in a number of cities in India. The ADEH now actively engages with a number of policy issues, campaigns for universal healthcare, and hosts meetings and conferences to push the agenda of ethical and rational healthcare. The Citizen-Doctor’s forum is a unique forum where a citizens charter has been evolved as a participatory process, and there are discussions between doctors and citizens on a number of issues including corruption.

The Indian example offers a reconceptualization of the doctor-patient and the doctor-market relationship. By recognizing and forming networks of ‘ethical doctors’ all over the country who are widely recognized as being non-corrupt, the initiative potentially unsettles the ‘norm’ and acceptability of corrupt practices. This happens not only in society at large, but also among the doctors as a profession. By simultaneously evolving Citizens Doctors Forums there is an attempt at complementing this with re-forging the doctor-patient relationship in more equal and trusting terms.

Such complex and multi-dimensional initiatives at the community-provider interface as well as at the level of the providers themselves, have the potential to create alternative discourses that have the potential to sustain alternative practices and arrangements that will make a true and lasting impact on corruption. Progress in anti-corruption activities needs to conceptualized as the evolution of truly democratic, just and caring systems of healthcare.

## Ethical issues

Not applicable.

## Competing interests

Author declares that he has no competing interests.

## Author’s contribution

RG is the single author of the paper.
